# First Molecular Confirmation of Equine Ocular *Setaria digitata* in China

**DOI:** 10.3390/vetsci8040055

**Published:** 2021-03-28

**Authors:** Feng Yu, Bo Liu, Shulei Chen, Ziwen Yi, Xianyong Liu, Yiping Zhu, Jing Li

**Affiliations:** 1 Equine Clinical Diagnostic Center, College of Veterinary Medicine, China Agricultural University, No. 2 Yuanmingyuan West Road, Beijing 100093, China; fengyu@cau.edu.cn (F.Y.); bliu2017@cau.edu.cn (B.L.); shuleichen20@163.com (S.C.); sy20203050871@cau.edu.cn (Z.Y.); 2 Department of Veterinary Parasitology, College of Veterinary Medicine, China Agricultural University, Beijing 100193, China; liuxianyong@cau.edu.cn

**Keywords:** horses, ophthalmology, setariasis, molecular diagnosis, China

## Abstract

A 5-year-old Mongolian mare (*Equus caballus* Linnaeus, 1758) was observed to have corneal opacity and excessive ocular discharge. An ophthalmic examination revealed a moving thread-like cylindrical worm in the anterior chamber of the right eye. The parasite was successfully removed surgically. The worm was observed under light microscopy and confirmed as *Setaria digitata* by 12S rRNA gene amplification and sequencing. Phylogenetic analysis demonstrated similarity with *Setaria digitata* in the National Center for Biotechnology Information (NCBI) GenBank database isolated from other Asian countries. This report is the first confirmed case of equine ocular setariasis by molecular diagnosis in China, which may indicate its presence in livestock and promote research on its epidemiology.

## 1. Introduction 

*Setaria* is a genus of roundworms (Nematoda: Filariodea), among which *Setaria digitata* is mainly found in Asia. *Setaria digitata* often affects cattle and buffalos, with common predilection site in peritoneal cavity [1]. Within its natural hosts, these nematodes are non-pathogenic in most cases. However, the parasite exhibits migratory behaviors in aberrant hosts including goats, sheep and horses [2]. The most common form of migration in infected horses is ocular migration [3,4]. Infected goats and sheep can experience cerebrospinal nematodiasis, which leads to lumbar paralysis and death [5]. Vectors of *S. digitata* include biting insects like mosquitoes (genera *Aedes, Culex, Anopheles, Hyrcanus* and *Armigeres*) [6]. Setariosis poses a serious threat to susceptible animals in tropical areas. When mosquitoes feed on the blood of infected hosts, they become infected with microfilariae, which develop into infective larvae (L3) inside the mosquitoes within 2 to 3 weeks. Infected mosquitoes transmit L3 to susceptible hosts during blood meals, after which it matures in the definitive host within 8–10 months [7].

Equine ocular setariasis is caused by *S. digitata*, *S. equina* or *S. marshalli*, with most cases reported by countries in south and southeast Asia [8,9,10,11]. According to a retrospective study conducted in India, 57% (138/242) of the regional cases of equine ocular disorders were diagnosed as ocular setariasis [12]. Infected horses displayed multiple ocular signs including lacrimation, photophobia, corneal opacity, conjunctivitis and blindness, especially when treatment was delayed [12,13,14]. Corneal edema, synechia, cataract and retinal detachments may occur with toxins released from worms in the anterior chamber [15]. 

*Setaria digitata* infecting horses in China has never been identified. This report is the first time *S. digitata* has been identified in the eye of an equine patient in China by molecular diagnosis. 

## 2. Materials and Methods

All procedures involving the horse in this study were carried out with a welfare license (AW62201202-2-1) issued by the Animal Care and Use Committee of the China Agriculture University in Beijing.

### 2.1. Case Report

**Figure 1:**
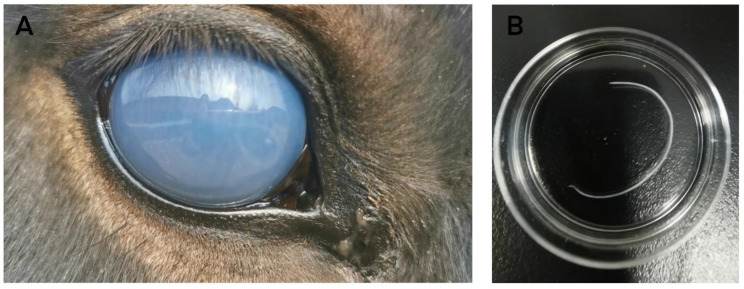
Corneal edema and ocular discharge in the right eye upon examination (**A**). Extracted *S. digitata* worm from the eye (**B**).

A 5-year-old Mongolian mare (*Equus caballus* Linnaeus, 1758) presented with blepharospasm, squinting and photophobia to the Equine Clinical Diagnostic Center of China Agricultural University. A gross ophthalmic examination identified a corneal opacity and ocular discharge in the patient’s right eye (Figure 1). Closer observation revealed a thread-like cylindrical worm, moving in a swirling motion, in the anterior chamber of the right eye. Microfilariae were not detected on a peripheral blood smear, and a complete blood count did not show major abnormalities. Surgical removal was chosen over exclusive medical treatment to remove the worm immediately. The mare was sedated with Xylazole (an α-2 agonist sedative) 1.1 mg/kg intravenously and was induced with and maintained on Tiletamine-Zolazepam 2.0 mg/kg intravenously. The right eye was flushed multiple times with 3% boric acid solution, and the cornea was desensitized with drops of proparacaine hydrochloride ophthalmic solution. Wrapped tightly in sterile medical gauzes and with 2 mm of its tip exposed, a #11 surgical blade was used to puncture open the anterior chamber of the right eye at the 6 o’clock position of the cornea, about 2 mm inside the limbus. The worm moved towards the puncture site and was easily removed with minimal aqueous leakage. The puncture site was left to heal with a 2 mm surgical lesion. Terramycin ophthalmic ointment was administered four times daily for one week as prophylaxis, and flunixin meglumine was injected intravenously at 1.1 mg/kg once daily for three days as an anti-inflammatory medication. The extracted worm was washed three times with phosphate buffer saline (PBS), observed under a light microscope, then preserved in 70% ethanol until its DNA was extracted. The ocular opacity completely cleared approximately 4 weeks after surgery. No other medications were prescribed considering the horse recovered without complications. 

### 2.2. Worm Identification

Light microscopy was used to identify the worm’s species based on referenced morphological criteria [16]. DNA was extracted from the worm by following the instructions specified in the genomic DNA extraction kit (TSINGKE, Beijing, China). The worm was digested with 20 uL Proteinase K and 200 uL distilled water in a 1.5 mL centrifuge tube. The tube was vortexed for 10 s, then 200 uL buffer gA1 was added. The tube was then vortexed for another 10 s. The sample incubated in warm water at 56 °C for 1 to 3 h. After DNA extraction, molecular diagnosis by Polymerase Chain Reaction (PCR) was initiated using the following primer sequence SDF: 5′-AGT CCT CCC TTG TTG CTG GT-3′ and SDR: 5′-GGG TGG TTT GTA CCC CTC CG-3′ [7]. A final PCR volume of 50 μL contained 25 μL 2XTSINGKE Master Mix, 1 μL of each forward and reverse primer and 1 μL of DNA template and 22 μL ddH2O. PCR amplification was performed under the following conditions: initial denaturation at 94 °C for 4 min for 1 cycle, denaturation at 94 °C for 80 s, annealing at 46 °C for 80 s, extension at 72 °C for 60 s for 39 cycles and final extension at 72 °C for 10 min. Amplified products were eletrophoresed on agarose gel and purified for further nucleotide sequences. The resulting sequences were compared with other filarial worms’ sequences stored in GenBank of the National Center for Biotechnology Information (NCBI) using the Basic Local Alignment Search Tool (BLAST) program. Available at: https://blast.ncbi.nlm.nih.gov/Blast.cgi (accessed on 12 January 2021). A multiple sequence alignment was obtained with the ClustalW program within MEGA X (Pennsylvania State University, State College, PA, USA). Phylogenetic analysis was then performed using MEGA X. For distance analysis, the Kimura 2-parameter model was used to construct the distance matrix and the tree was inferred from this using the Maximum Likelihood approach. Bootstrap resampling (1000 pseudoreplicates) was performed, and a bootstrap consensus tree was produced. 

## 3. Results and Discussion

**Figure 2:**
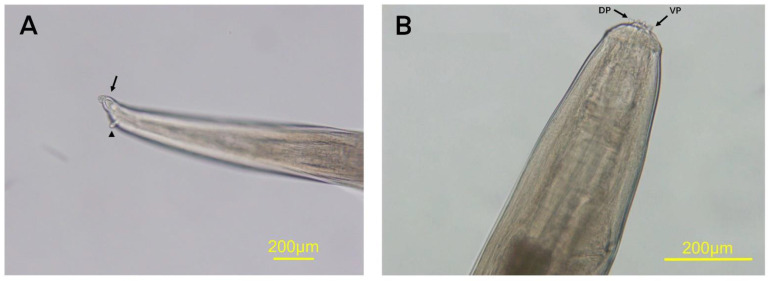
Posterior end of female adult of *Setaria digitata* has spherical terminal knob (arrow) and lateral appendages (arrowhead) (**A**). Anterior region of *S. digitata* shows the dorsal projection (DP) and ventral projections (VP) on peribuccal crown (**B**).

The extracted worm was milky white in color, thread-shaped, tapered at both ends and 7.2 cm long. The tapering posterior end had a spherical knob, which is characteristic of a female *S. digitata* (Figure 2). The anterior end was round with two equal prominences on the peribuccal crown (Figure 2). Varying lengths of *S. digitata* from cattle and horses were reported [16,17,18,19].

**Figure 3:**
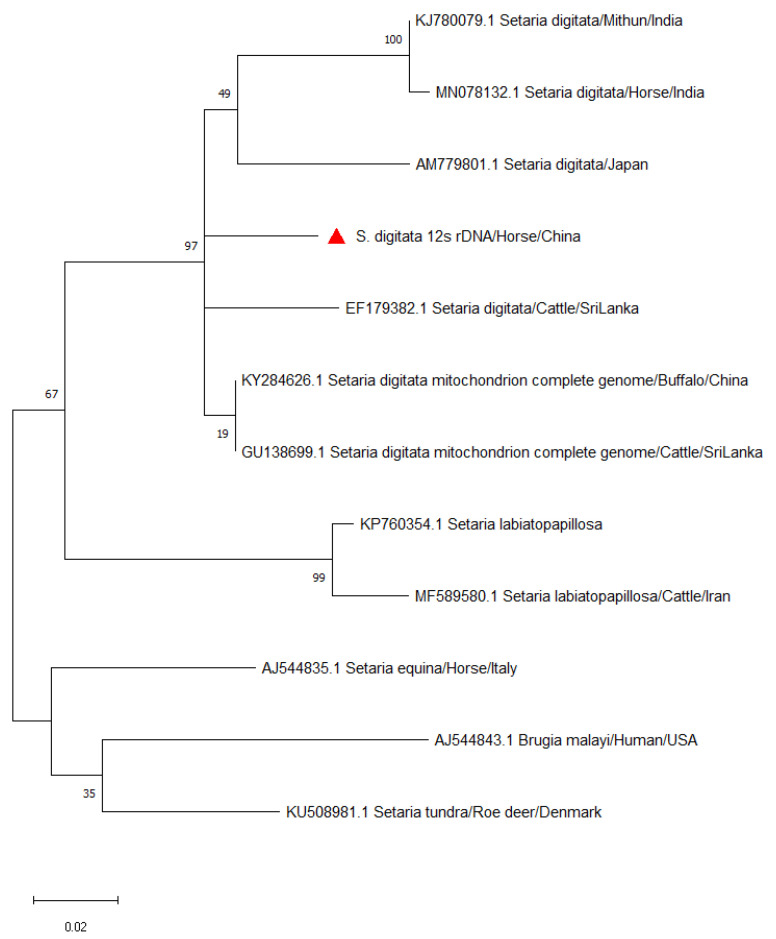
A mind-point rooted phylogenetic tree, inferred from 12SrDNA nucleotide sequences using the Maximum Likelihood method in MEGA X after Kimura-2 correction. Scale bar indicates the proportion of sites changing along each branch. Accession numbers of all sequences used for tree analyses are given. The sequence generated in this study was marked as red rectangular.

Even though morphology has been traditionally used to identify and differentiate *Setaria* spp. [7], it was reported to be inadequate since *S. digitata* and its congeners, including *S. digitata*, *S. equina*, *S. labiatopapillosa* and *S. cervi,* are similar in morphology [19]. Instead, this study used a nucleic acid-based detection method to confirm the filarial species isolated from the mare. Gel electrophoresis produced a 209 bp band, and the sample was confirmed as *S. digitata* when compared against other sequences in the NCBI GenBank database using BLAST. The genetic sequence extracted from the worm was 99% similar to that of *S. digitata* isolated from cattle and buffalos in Sri Lanka and from buffalos in China [20,21]. The mind-point rooted phylogenetic tree further confirmed that the *S. digitata* isolated from this study belongs to the same major clade isolated from Japan, India and Sri Lanka. *S. equina* and *S. tundra* formed in a different cluster. *Setaria digitata* and *S. labiatopapillosa* appear to be sister species with a bootstrap value of 67% (Figure 3). The 12S rDNA sequence was a useful marker for phylogenetic analysis due to its slow evolution compared to other protein coding mitochondrial genes [8,20]. Phylogenetic analysis of *S. digitata* indicated that the isolate sequences from different host species produced a closely related clade with high bootstrap support within the *Setaria* genus. Minor nucleotide sequence variations were observed. The current findings imply that the isolates of *S. digitata* detected worldwide have the same molecular characteristics [19].

Different medical therapies have been proposed to treat equine ocular setariasis to avoid complications of surgical treatments such as phthisis bulbi, corneal edema and prolapse of iris. Medical therapies have their inert disadvantages. For example, intraocular reaction to the dead worm body may lead to more severe damage to the intraocular structure [22,23]. Raziq, SA (1989) suggested diethylcarbamazine citrate as a treatment option for microfilaremia, but it was not effective against adult parasites in the eye [24]. Another study demonstrated that ivermectin resolved microfilaremia seven days post-treatment; however, the adult worms took longer to treat [25]. Given the limitations of medical management, surgical treatment was chosen for the immediate removal of the adult worm with minimal risk. In most *S. digitata*-related ocular cases, only one worm was retrieved from horses even though two worms can be encountered [19,26]. There is no confirmed association between length or load of worms in eyes and the severity of ocular disease. Also, ocular lesions healed well in most cases [8,18,19]. 

*Setaria digitata* was mostly reported in tropical Asian countries, where the vectors are prevalent [5,8,9,11,17,19]. South Korea was the only high latitude non-tropical country where *S. digitata* was reported in horses [18,26]. In this study, *S. digitata* was identified and confirmed by a molecular method in a horse on a farm located in northern China, where the latitude was close to South Korea. Interestingly, the case occurred during this region’s coldest time of the year (December), which indicates that the mare was infected long before December, when the vectors were active. The occurrence of this incidental case is likely due to the presence of cattle farms in the neighborhood. Therefore, this finding suggests that cattle in northern China may carry *S. digitata*, and further investigation of its prevalence will be worthwhile. In 2017, *S. digitata* was first identified by molecular method in the cadavers of buffalos in southern China, yet not in live buffalos or cattle [21]. Setariasis is a well-recognized ophthalmological disorder in equine, with *S. digitata* being reported as the most common cause [27]. *Setaria digitata* is considered a major health hazard in animals, with detrimental economic impact on the owners. Therefore, the findings from this study warrant further epidemiological investigation of *Setaria digitata* in China, especially in the natural hosts. 

## Data Availability

Data sharing not applicable.
